# Application of Photocrosslinkable Wet-Adhesive Composite Hydrogel Adhesive in Bladder Injury Repair

**DOI:** 10.3390/bioengineering13091047

**Published:** 2026-09-09

**Authors:** Kaipeng Bi, Ziyan An, Zheng Wang, Guanqiu Chen, Zhoujie Ye, Jinpeng Shao, Zhouyang Fu, Qi Cai, Shuwei Xiao, Weijun Fu

**Affiliations:** 1 School of Medicine, Nankai University, Tianjin 300071, China; 1120230910@mail.nankai.edu.cn (K.B.); 1120251181@mail.nankai.edu.cn (Z.Y.); 2120241897@mail.nankai.edu.cn (Q.C.); 2 Senior Department of Urology, Chinese PLA General Hospital, Beijing 100039, China; wz995731879@163.com (Z.W.); chenguanqiu2010@126.com (G.C.); shaojp0629@163.com (J.S.); fuzhouyang888@163.com (Z.F.); 3 Department of Urology, Beijing Shijitan Hospital, Capital Medical University, Beijing 100038, China; anziyan301@163.com; 4 Department of Urology, Air Force Medical Center, Beijing 100142, China

**Keywords:** bladder injury, hydrogel, adhesive, bladder tissue engineering, regenerative medicine

## Abstract

Medical adhesives have been extensively applied for skin wound repair and hemostasis, while the dynamically humid and complex intraperitoneal environment imposes stringent requirements on tissue adhesives. Few studies have reported the application of biological adhesives for bladder defect repair in urological practice. The GSF composite hydrogel adhesive, composed of gelatin methacryloyl (GelMA), silk fibroin methacryloyl (SilMA), and polyether F127 diacrylate (F127DA), was previously fabricated with favorable injectability, appropriate viscosity and superior mechanical toughness. To further confirm the repair efficacy, GSF was characterized in vitro for the adhesive properties and structural stability under dynamically humid conditions. Corresponding in vivo experiments were conducted on rat models with graded bladder rupture and partial bladder defects to further assess the repair performance of GSF. Results showed that GSF hydrogel retained robust interfacial adhesion and intact structural stability for 8 days under dynamically simulated humid intraperitoneal conditions and cyclic urine filling-emptying environments. GSF exhibited rapid bonding capacity and effectively shortened the operation time. Under moderate bladder injury conditions, the wound closure time of the GSF group was only 28.3% of that of the suture group in cystotomy experiment and 34.5% in cystectomy experiment. It achieved satisfactory repair of mild-to-moderate bladder ruptures and partial bladder defects. Postoperative histological and functional evaluations confirmed that GSF hydrogel facilitated bladder structural reconstruction and functional recovery. These findings highlight the significant potential of GSF hydrogel adhesives for repairing and regenerating injured bladders. Future applications of GSF will extend to adhesive repairs of organs within the humidity conditions of the abdominal cavity.

## 1. Introduction

Severe bladder trauma, bladder diverticula, and other related conditions are commonly treated through surgery, with absorbable sutures often being the preferred choice [1,2]. The bladder, as a urine storage organ, requires precise suturing techniques, and the presence of sutures may interfere with optimal healing [3]. In contrast, adhesives offer several advantages over sutures, including simplicity, faster application, reduced tissue damage, and prevention of fluid leakage [4]. However, current tissue adhesives used clinically exhibit significant drawbacks. Cyanoacrylate glue, for instance, suffers from poor biocompatibility and low flexibility, while fibrin adhesives have limited bonding strength and mechanical properties [5]. Consequently, ongoing research focuses on developing novel tissue adhesives with improved properties.

Hydrogels, which are polymeric crosslinked networks with high water content, have the potential to mimic the extracellular matrix. Due to their rich functional groups, hydrogels can form interactions both through chemical bonds and via hydrogen bonding and van der Waals forces, facilitating bioadhesion [6]. Yang et al. developed an injectable hydrogel based on ε-polylysine and a poly(ethylene glycol) derivative, designed for emergency hemostasis and wound repair. The NHS esters in this hydrogel spontaneously react with amine groups, forming a cross-linked gel that covalently bonds to tissue surfaces, ensuring robust adhesion [7]. Xie et al. created an injectable hydrogel adhesive for treating diabetes-induced skin injury, where adhesion is achieved through hydrogen bonding between catechol-modified carboxymethyl cellulose and tannic acid [8]. However, the majority of research in tissue adhesives has focused on skin applications, with limited exploration of their use in hollow organs. Hollow organs, typically located in the humid environment of the abdominal cavity and designed to store liquids, present additional challenges due to their complex internal conditions, demanding more advanced adhesives.

**Scheme 1:**
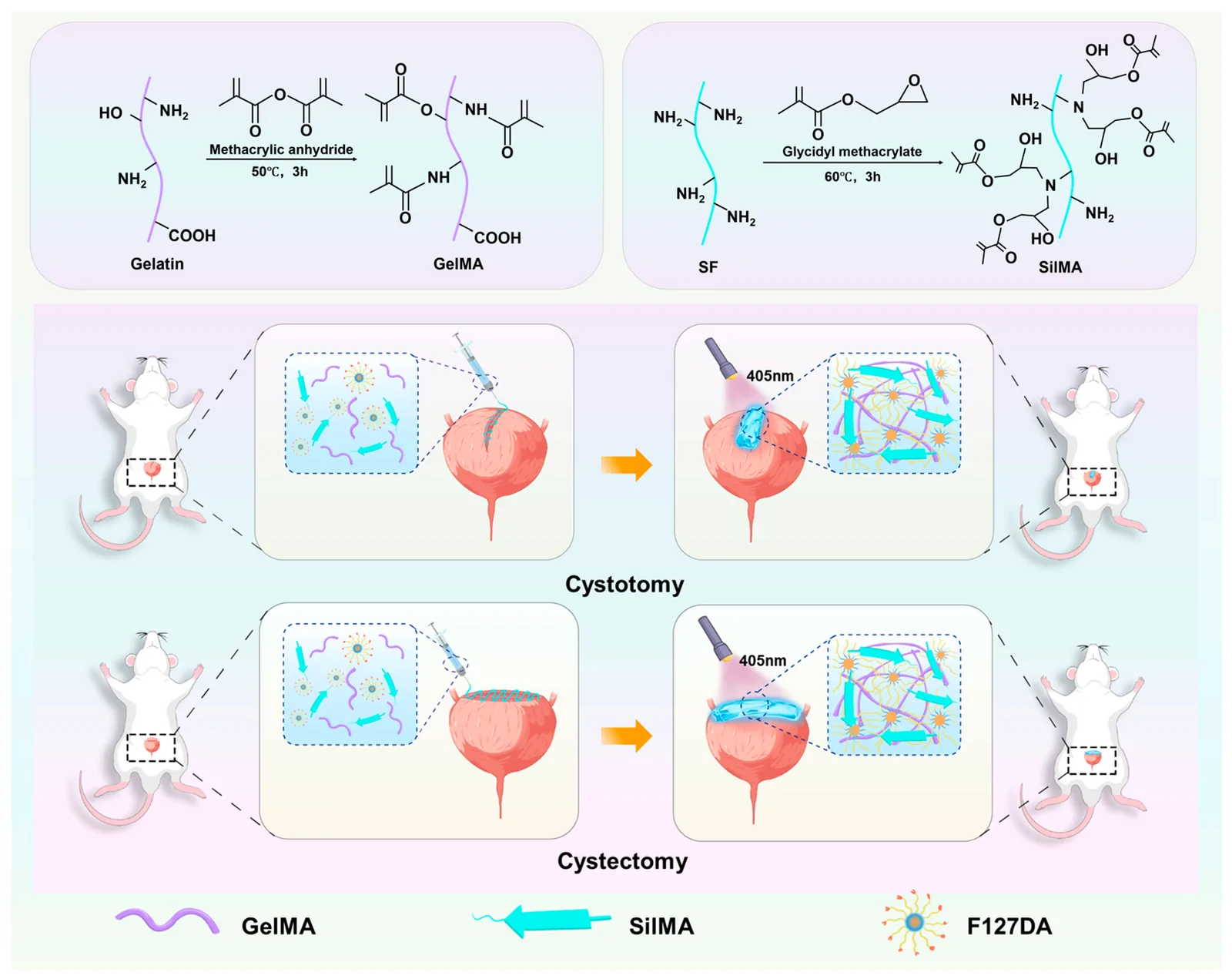
Chemical reaction principle of GelMA and SilMA and the application of GSF composite hydrogels in bladder injury.

In our previous work, a composite hydrogel adhesive (GSF) consisting of gelatin methacryloyl (GelMA), silk fibroin methacryloyl (SilMA), and polyether F127 diacrylate (F127DA) was successfully fabricated. The GSF hydrogel forms a synergistic polymer network via photo-induced covalent crosslinking and non-covalent hydrogen-bonding interactions, thereby integrating favorable injectability, superior mechanical performance, and robust bioadhesive properties [9]. In the present study, the GSF hydrogel was further characterized in terms of the adhesive and sealing performance under dynamically humid conditions. Its therapeutic efficacy was rigorously evaluated in animal models to support its potential clinical application in bladder injury repair (Scheme 1).

## 2. Materials and Methods

### 2.1. Preparation and Characterization of GelMA and SilMA

GelMA and SilMA were synthesized according to previous reports with slight modifications [10,11]. Briefly, 10 g of porcine skin-derived gelatin type A (Sigma-Aldrich, St. Louis, MO, USA) was dissolved in 100 mL of phosphate-buffered saline (PBS) at 50 °C to obtain a 10% (*w*/*v*) homogeneous solution. Then, 8 mL of methacrylic anhydride (Sigma-Aldrich, St. Louis, MO, USA) was added dropwise to the gelatin solution. The reaction was conducted in the dark at 50 °C for 3 h. GelMA was isolated via dialysis of the reaction mixture and subsequent freeze-drying.

Silk fibroin was obtained by degumming cocoons in a 0.05 M Na_2_CO_3_ solution. The silk fibroin was dissolved in a 9.3 M LiBr (Sigma-Aldrich, St. Louis, MO, USA) solution. Subsequently, this solution was reacted with 424 mM glycidyl methacrylate (GMA, Sigma-Aldrich, St. Louis, MO, USA) at 60 °C for 3 h. SilMA was isolated via dialysis of the reaction mixture and subsequent freeze-drying.

Fourier transform infrared spectroscopy (FTIR, Bruker, Ettlingen, Germany) confirmed the successful modification of GelMA and SilMA. Spectra were acquired from 400 to 4000 cm^−1^ at a resolution of 4 cm^−1^.

### 2.2. Preparation of GSF Hydrogel

F127DA and lithium phenyl (2,4,6-trimethylbenzoyl) phosphinate (LAP) were sourced from EFL-tech Co., Ltd. (Suzhou, China). GelMA, SilMA, and F127DA were dissolved in PBS containing 0.25% (*w*/*v*) LAP to achieve a final concentration of 5% (*w*/*v*) GelMA, 10% (*w*/*v*) SilMA, and 5% (*w*/*v*) F127DA. The precursor solution of the GSF composite hydrogel, containing 0.25% (*w*/*v*) LAP, was irradiated with 405 nm light for cross-linking, resulting in the formation of the GSF composite hydrogel. The GS hydrogel (10% SilMA and 5% GelMA), as well as the pure 10% SilMA, was used to verify the characteristics of the GSF hydrogel. The contents of the three hydrogels were determined based on our previous systematic studies and empirical optimization [9].

### 2.3. Scanning Electron Microscope (SEM)

After being freeze-dried, three groups of hydrogel samples (n = 3) were subjected to brittle fracture in liquid nitrogen to expose their cross-sections. The morphology was observed using scanning electron microscopy (Zeiss, Oberkochen, Germany) after spraying the cross-sections of the samples with gold particles. For each hydrogel sample, three visual fields possessing uniform pore characteristics were randomly selected. The pore size was quantified from SEM micrographs using ImageJ software (Version 1.53).

### 2.4. Degradation Test

Three groups of cylindrical hydrogel samples (diameter = 10 mm, height = 5 mm, n = 40, 5 per time point) were prepared to evaluate the degradation performance of the hydrogels. The hydrogels were immersed in simulated human urine (Solarbio, Beijing, China) at 37 °C with a shaking speed of 60 rpm. At various time points, the urine was aspirated, and the remaining hydrogel samples were freeze-dried. The dry weight of the hydrogels was measured to plot the degradation curves.

### 2.5. Mechanical Property

Tensile tests were performed on a universal mechanical testing machine (Instron, Norwood, MA, USA). Hydrogel samples (n = 4) from each group were prepared in a dumbbell shape (gauge width = 2 mm, gauge thickness = 2.5 mm) and stretched to fracture at a rate of 5 mm/min. The Young’s modulus was determined by the slope of the stress–strain curve.

### 2.6. Biocompatibility of the Hydrogels

Adipose-derived stem cells (Otwo Biotech, Shenzhen, China) were used to verify the biocompatibility of GSF hydrogel. Three groups of hydrogel samples were prepared and immersed in complete DMEM medium at 37 °C for 24 h. Cell viability was then assessed using a Live/Dead cytotoxicity kit (Invitrogen, Carlsbad, CA, USA). Specifically, the samples were stained with calcein-AM and ethidium homodimer-1 on days 1 and 3, respectively, and imaged with a fluorescence microscope. Cell viability was calculated by dividing the number of live cells (green) by the total number of cells. Six representative images from each group were included for statistical analysis. The experiment was performed in triplicate.

### 2.7. Wound Closure Test

Based on ASTM F2458-05(2024) standards, porcine bladder tissue was used to evaluate the wound closure adhesion strength of the hydrogels. A moistened tissue sample (20 mm × 50 mm, n = 4) was incised at the center to simulate a wound. Subsequently, 200 µL of the hydrogel precursor solution was injected into the incision. The left and right adhesion areas were symmetrical, each with a bonding area of 5 × 20 mm. After UV irradiation, the two tissue flaps were pulled apart in the shear direction at a rate of 10 mm/min until complete hydrogel detachment. Wound closure adhesion strength was calculated based on the maximum stress and the bonding area.

### 2.8. Cyclic Tensile Test

The incised porcine bladder tissue was repaired with GSF hydrogel and then underwent a cyclic tensile test at a crosshead speed of 10 mm/min with a maximum strain of 100% for five cycles. Untreated bladder tissue served as the control group (n = 4).

### 2.9. Wet Adhesion and Sealing Property of GSF Hydrogel

Fresh porcine bladder was surgically incised to create a 3 cm rupture. To simulate the state of moisture, the porcine bladder was fully submerged in water and subsequently removed. A 1 mL volume of the GSF hydrogel precursor solution was applied to the incision, followed by irradiation with 405 nm light for 1 min to induce cross-linking. The bladder was immersed in PBS solution. A Silicone tube was used to connect the bladder, with the other end connected via a peristaltic pump (LongerPump, Baoding, China) to a bottle filled with simulated human urine. The entire apparatus was maintained at 37 °C. Simulated human urine was infused into the bladder at a rate of 60 mL/h. The bladder was emptied every 4 h, with four cycles per day, and remained filled for 8 h. Wet adhesion and sealing effects were evaluated at 1, 2, 4, 8 days after the procedure (n = 3).

### 2.10. Animal Experiment Design

A total of 72 eight-week-old Sprague–Dawley (SD) rats (SPF Beijing Biotechnology, Beijing, China) were enrolled in this study and assigned to two experimental models, including partial cystotomy for the simulation of bladder rupture and partial cystectomy for the establishment of partial bladder defects. Each model group was further subdivided into mild, moderate, and severe injury categories, which were treated with either GSF hydrogel repair or suture repair (n = 6 per subgroup). Four weeks post-operation, bladder ultrasound and urodynamic assessments were conducted, followed by sacrifice for histological analysis.

### 2.11. Bladder Injury Modeling and Repair Scheme

For the surgical procedures, SD rats were anesthetized using a small animal anesthesia machine. After skin preparation and disinfection, a midline incision was made to fully expose the bladder. In the cystotomy procedure, mild, moderate, and severe injuries were created by incising 25%, 50%, and 75% of the bladder wall along the midline longitudinally, corresponding to lengths of 5 mm, 10 mm, and 15 mm along the bladder circumference, respectively. In the cystectomy procedure, 10%, 20%, and 30% of the bladder wall at the top of the bladder were excised, representing mild, moderate, and severe injury. The excised bladder tissue was quantified using ImageJ software (Version 1.53). In the experimental group, GSF hydrogel was applied to repair the bladder, while the control group received sutures. The GSF composite hydrogel precursor solution was applied to the bladder wound, followed by 405 nm light irradiation for 1 min to promote cross-linking (Video S1). Simple interrupted sutures were used in the control group with 6-0 absorbable sutures (Video S2). The time required for bonding and suturing was recorded.

### 2.12. Ultrasound Examination

Ultrasound examination was conducted 4 weeks post-surgery using a small animal ultrasound system. Both transverse and sagittal views of the rat bladder were captured to assess its morphology and detect any stones.

### 2.13. Urodynamic Examination

Urodynamic testing was also performed 4 weeks after surgery. Following anesthesia, the bladder was surgically exposed, and a PE-50 tube was inserted into the bladder and secured with a purse-string suture. The open end of the PE-50 tube was connected to a pressure measurement module via a three-way stopcock. During continuous infusion of warm sterile saline at a rate of 200 μL/min, bladder pressure and capacity were recorded. Key urodynamic parameters, including maximal bladder capacity (V), peak micturition pressure (P), and bladder compliance (V/P), were analyzed.

### 2.14. Histological Evaluation

At 4 weeks post-surgery, the rat was anesthetized, and the bladder was exposed for morphological evaluation and photographic documentation. The bladder was then excised, cleaned, and fixed in 4% paraformaldehyde. Following fixation, tissues were dehydrated through a gradient of alcohols, embedded in paraffin, and sectioned into 4 μm thick slices. Histological staining was performed using hematoxylin and eosin (H&E) and Masson’s trichrome.

Tissue sections were incubated overnight at 4 °C with rabbit polyclonal antibodies targeting α-smooth muscle actin (α-SMA) and CD31. Afterward, sections were incubated with species-specific FITC-conjugated secondary antibodies, and the nuclei were counterstained with DAPI. Images were acquired using a pathology slide scanner, and six random fields from six sections were selected to analyze the fluorescence area for α-SMA and CD31 using ImageJ software (Version 1.53).

To minimize subjective bias and ensure the objectivity and reliability of the results, all image acquisitions and subsequent quantitative analyses for immunofluorescence assessments were performed in a blinded manner. Two independent researchers who were unaware of the experimental grouping and treatment information separately conducted image collection and quantitative statistics. The obtained data were cross-checked to avoid potential analytical bias.

### 2.15. Statistical Analysis

Statistical analysis and data visualization were conducted using GraphPad Prism 9.0. All data were first assessed for normality and homogeneity of variance. Quantitative data are presented as the mean ± standard deviation (Mean ± SD). One-way ANOVA with Tukey’s post hoc test was used for comparisons among hydrogel groups, while two-way ANOVA followed by Sidak’s post hoc test was applied for comparisons among animal groups. Statistical significance was defined as *p* < 0.05.

## 3. Results

### 3.1. Preparation and Characterization of GSF Hydrogels

**Figure 1:**
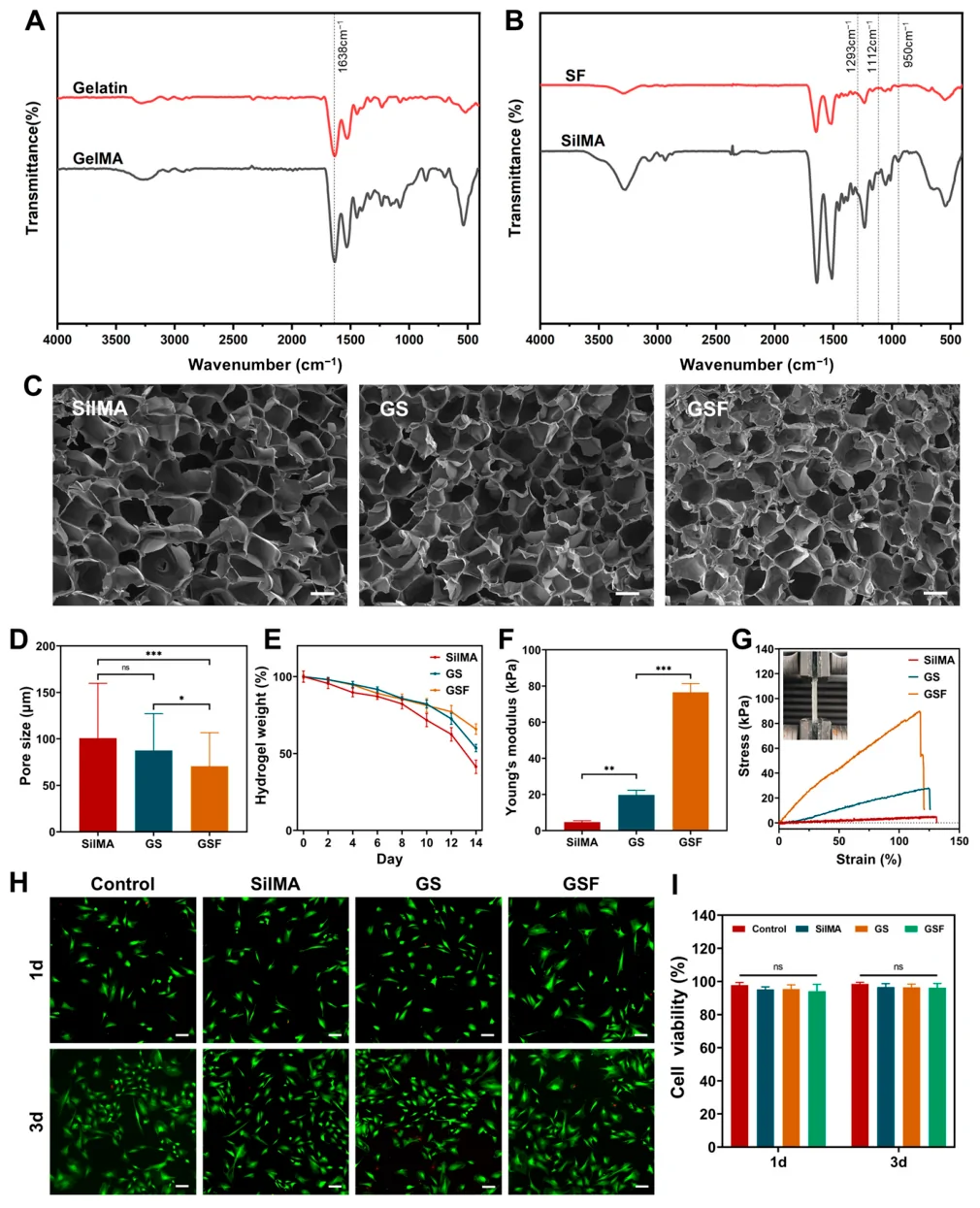
Preparation and characterization of GSF hydrogels. FTIR spectroscopy for GelMA (**A**) and SilMA (**B**). (**C**,**D**) Cross-sectional SEM images and pore size of GSF hydrogel. (**E**) Degradation curve of SilMA, GS, and GSF hydrogels in 14 days. (**F**,**G**) Mechanical properties of SilMA, GS, and GSF hydrogels. (**H**,**I**) Biocompatibility of SilMA, GS, and GSF hydrogels. (Scale bar: 100 μm, *p*-values are calculated using the one-way ANOVA followed by Tukey’s post hoc test, ns, *p* > 0.05, * *p* < 0.05, ** *p* < 0.01, *** *p* < 0.001).

FTIR spectroscopy confirmed the successful synthesis of GelMA and SilMA. The FTIR spectrum of gelatin contained peaks at 1638 (amide I), attributable to the C=O bond stretching vibrations. The peak intensity in the amide I region increased after MA modification, attributable to the stress vibration of the methacrylate group’s C=C bond (Figure 1A). A weak spectral band in SilMA groups can be observed at 1293 cm^−1^, which is attributed to the CHOH stretching vibration of the alcohol group after the opening of the epoxy group of the GMA. Additionally, the minor spectral changes were at 950 cm^−1^ and 1112 cm^−1^, representing CH_2_ wagging stretching of the methacrylate vinyl group (Figure 1B).

Scanning electron microscopy revealed that SilMA and GS hydrogels possess uniformly distributed pores. The GSF hydrogel displayed a denser porous structure with heterogeneous pore sizes, and its pore size (70.53 ± 36.15 μm) was significantly smaller than those of SilMA (100.67 ± 59.11 μm, *p* < 0.001) and GS hydrogels (87.52 ± 39.73 μm, *p* < 0.05) (Figure 1C,D). Compared with SilMA and GS hydrogels, GSF hydrogel exhibited a slower degradation rate, with approximately 65% remaining after 14 days in simulated human urine, attributed to its higher crosslinking density (Figure 1E). The mechanical properties of the hydrogels were evaluated through tensile tests. The stress–strain curves indicated that the incorporation of GelMA and F127DA significantly enhanced the mechanical properties of the hydrogels. The Young’s modulus of SilMA, GS, and GSF hydrogels were 4.72 ± 0.69 kPa, 19.65 ± 2.62 kPa, and 76.52 ± 4.85 kPa, respectively (Figure 1F,G). Biocompatibility was verified by culturing ADSCs in the hydrogel extracts. Cell viability on days 1 and 3 showed that all three hydrogels exhibited no significant cytotoxicity compared with the control group (Figure 1H,I).

### 3.2. Adhesive Properties of GSF Hydrogel

**Figure 2:**
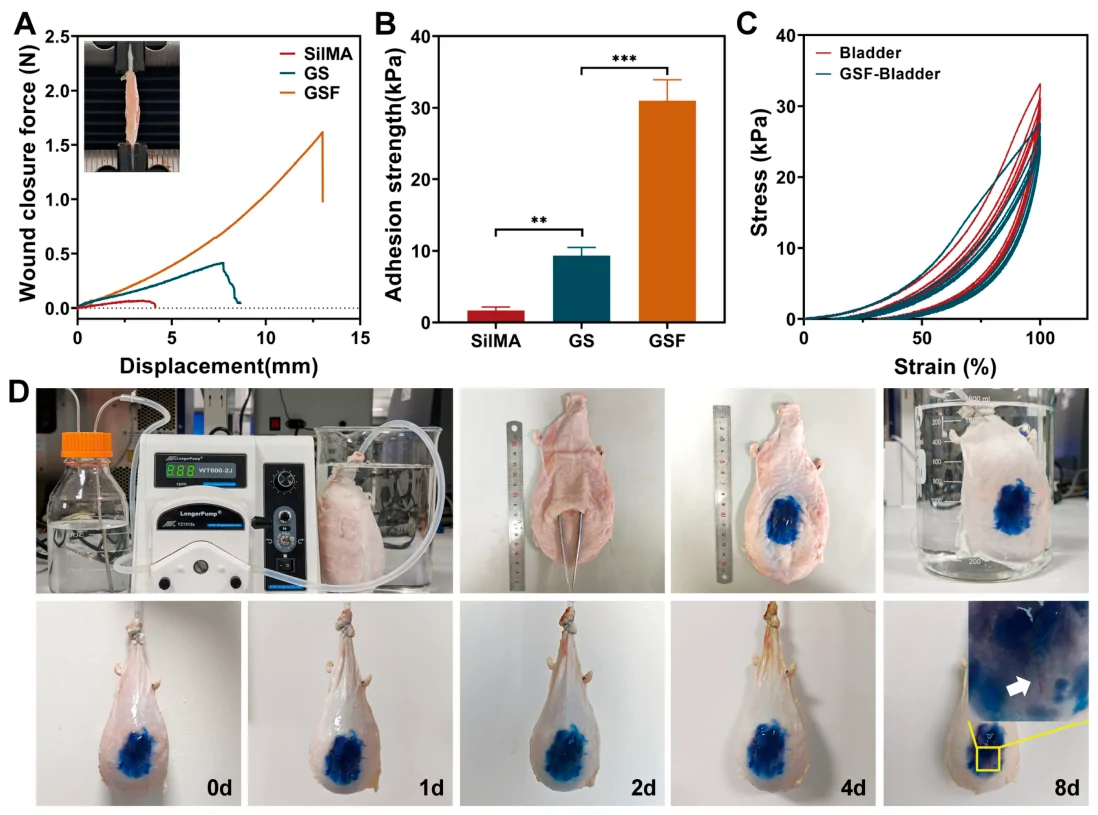
Adhesive properties of GSF hydrogel. (**A**,**B**) Wound closure test. (**C**) cyclic tensile test of bladder and GSF-repaired bladder. (**D**) Wet adhesion and sealing properties of GSF Hydrogel. (*p*-values are calculated using the one-way ANOVA followed by Tukey’s post hoc test, ** *p* < 0.01, *** *p* < 0.001).

Wound closure test showed that the GSF hydrogel could generate a closure force exceeding 1.5 N on porcine bladder tissue (Figure 2A). The adhesive strength of the GSF hydrogel was 30.99 ± 2.92 kPa, significantly higher than that of SilMA (1.68 ± 0.47 kPa, *p* < 0.001) and GS (9.32 ± 1.17 kPa, *p* < 0.01) (Figure 2B).

Cyclic tensile test at 100% strain was performed (Figure 2C). Bladder tissue showed a decrease in stress after the first cycle, reflecting the contraction and relaxation properties of bladder smooth muscle. Compared with bladder tissue, the GSF-repaired bladder exhibited a larger hysteresis loop, reflecting energy dissipation from the breakage of unstable chemical bonds like hydrogen bonds in the adhesive region.

Bladder injury adhesion and repair experiment in vitro was conducted to evaluate the adhesion and sealing performance of the GSF hydrogel under dynamic wet conditions (Figure 2D). GSF hydrogel exhibited strong adhesion to the porcine bladder surface. Under dynamic wet conditions with cyclic filling and emptying, it maintained effective sealing performance after 8 days.

### 3.3. Efficacy of Bladder Injury Repair

**Figure 3:**
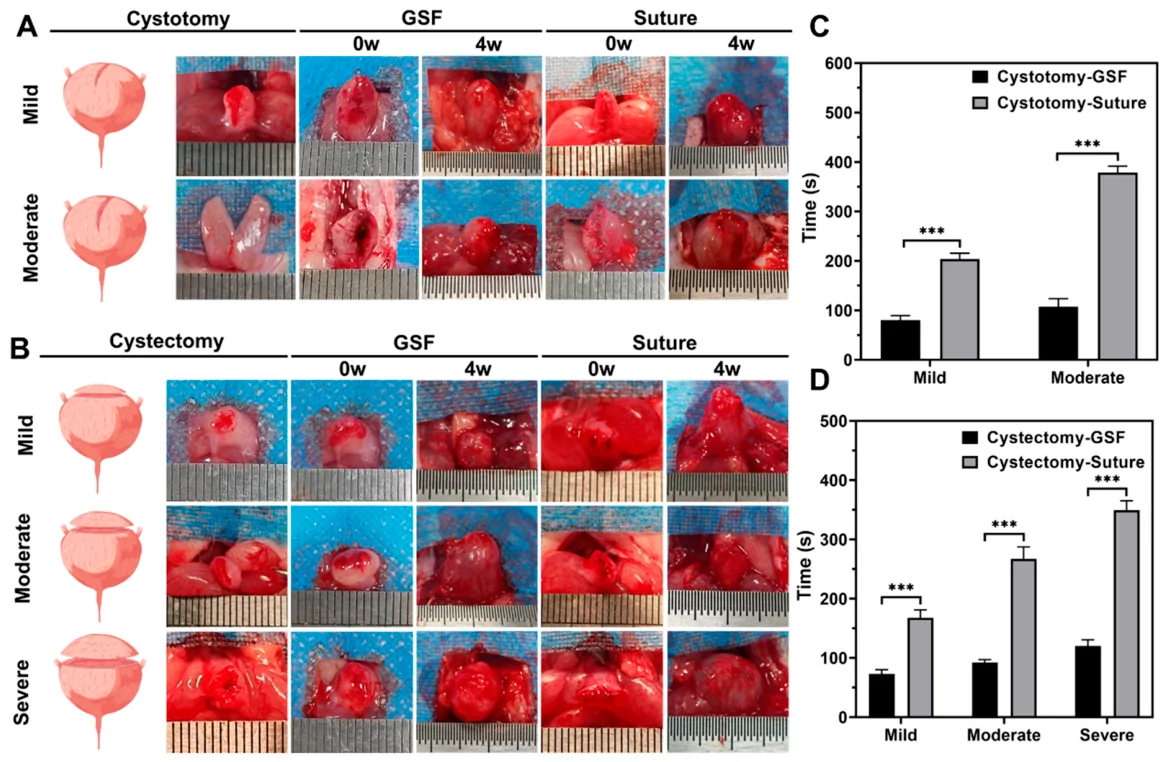
Gross bladder morphology following injury repair and comparison of operative repair time. (**A**,**B**) Construction of the bladder injury model and postoperative morphological assessment. (**C**,**D**) Operative time required for adhesive bonding and suture repair in bladder injury experiments. (*p*-values are calculated using the two-way ANOVA followed by Sidak’s post hoc test, *** *p* < 0.001).

To assess the efficacy of GSF hydrogel in bladder injury repair, the experiment was divided into two parts, with animal models of bladder cystotomy and cystectomy created to simulate varying degrees of injury (Figure 3A,B). In the severe cystotomy experiment, five rats in the GSF group and two rats in the suture group died within three days after the surgery. Therefore, the data were not included in the statistics. The remaining groups all survived until four weeks after the surgery. The time required for adhesive bonding was compared to suturing during the repair process (Figure 3C,D). In the cystotomy experiment, the time for adhesion was 80.17 ± 9.06 s for mild injury, whereas suturing took 203.67 ± 11.93 s (*p* < 0.001). For moderate injury, adhesion took 107.17 ± 16.30 s, while suturing took 378.67 ± 12.86 s (*p* < 0.001). In the cystectomy experiment, adhesion time was 73.33 ± 6.86 s for mild injury, compared to 167.83 ± 13.36 s for suturing (*p* < 0.001). For moderate injury, the adhesion time was 92.33 ± 4.63 s, while suturing took 267.33 ± 20.10 s (*p* < 0.001). For severe injury, adhesion took 120.33 ± 10.29 s, while suturing took 349.67 ± 15.55 s (*p* < 0.001).

Morphological assessment was conducted at 4 weeks post-surgery. In the suture group, significant tissue adhesion was evident, with the bladder appearing stretched. In contrast, less tissue adhesion was observed in the GSF group. Upon careful separation of the adhesive tissue, the bladder shape in the suture-repaired group appeared irregular, with some residual sutures visible. The bladder repaired with GSF maintained a normal shape (Figure 3A,B).

### 3.4. Ultrasound Examination

**Figure 4:**
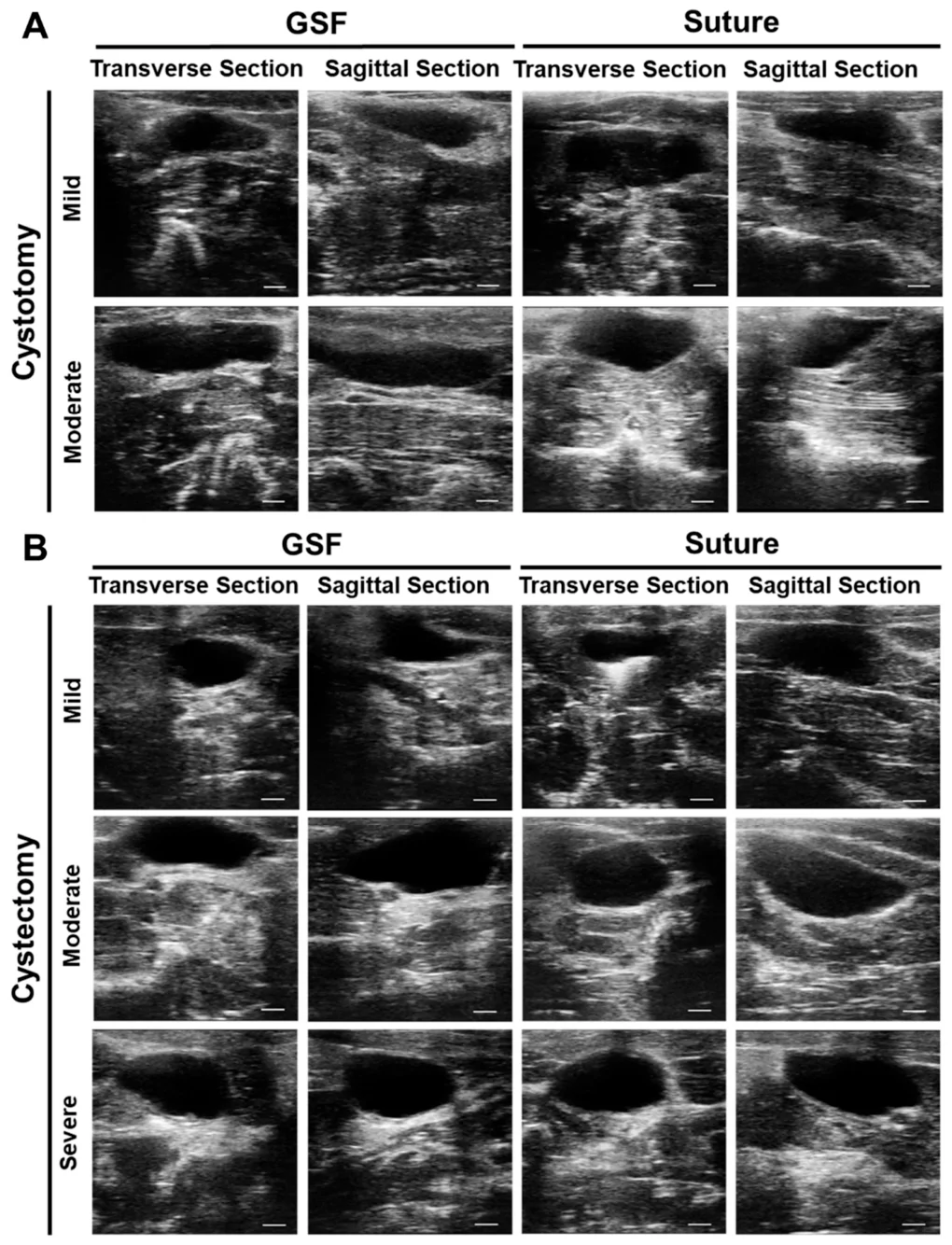
Bladder ultrasound examination at 4 weeks post-surgery. Bladder repair after cystotomy injury (**A**) and partial cystectomy injury (**B**). (Scale bar: 2 mm).

In terms of bladder ultrasound examination, the bladders in the cystotomy experiment appeared fuller. In the cystectomy experiment, part of the bladder wall was inwardly depressed. Both the GSF and suture-repaired bladders were well-filled, with no significant morphological differences observed between the two groups (Figure 4).

### 3.5. Urodynamic Examination

**Figure 5:**
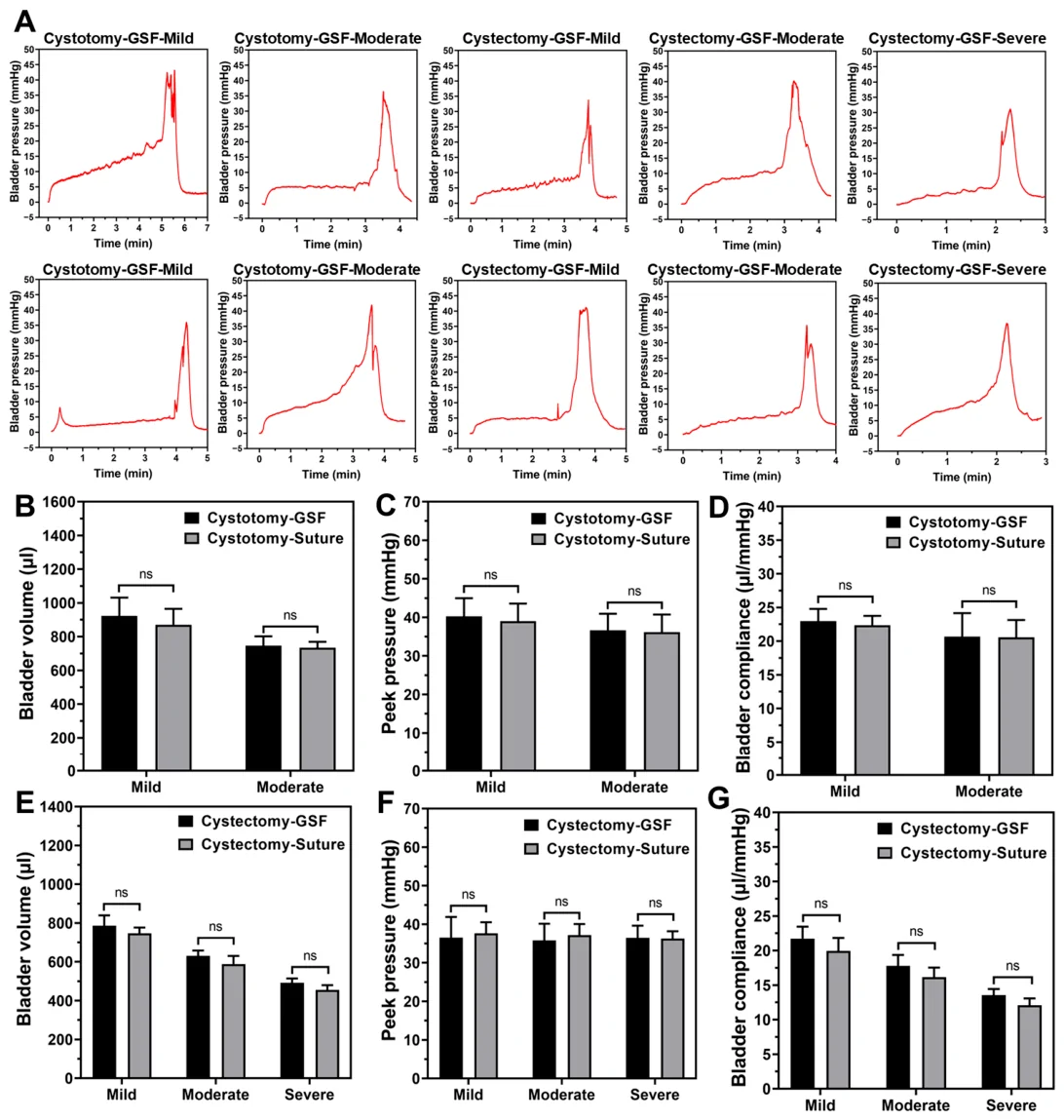
Urodynamic analysis and quantitative evaluation of urodynamic parameters at 4 weeks post-surgery. (**A**) Representative images of urodynamic examination. (**B**–**D**) Quantitative analysis of bladder volume, peak pressure, and bladder compliance in cystotomy experiments. (**E**–**G**) Quantitative analysis of bladder volume, peak pressure, and bladder compliance in cystectomy experiments. (*p*-values are calculated using the two-way ANOVA followed by Sidak’s post hoc test, ns, *p* > 0.05).

Urodynamic examination was conducted 4 weeks post-bladder repair to assess bladder capacity, maximum bladder pressure, and bladder compliance (Figure 5). In the cystotomy experiment, for the GSF-mild group, bladder capacity was 923.01 ± 108.62 μL, maximum bladder pressure was 40.27 ± 4.69 mmHg, and bladder compliance was 22.99 ± 1.80 μL/mmHg. In the suture-mild group, bladder capacity was 871.09 ± 93.83 μL, maximum bladder pressure was 39.01 ± 4.56 mmHg, and bladder compliance was 22.37 ± 1.38 μL/mmHg. No statistically significant differences were found between the GSF-mild and suture-mild groups in terms of bladder capacity, maximum bladder pressure, and bladder compliance. In the GSF-moderate group, bladder capacity was 746.70 ± 54.75 μL, maximum bladder pressure was 36.68 ± 4.26 mmHg, and bladder compliance was 20.68 ± 3.48 μL/mmHg. In the suture-moderate group, bladder capacity was 734.15 ± 35.07 μL, maximum bladder pressure was 36.18 ± 4.55 mmHg, and bladder compliance was 20.54 ± 2.60 μL/mmHg. Again, no statistically significant differences were observed between the GSF-moderate and suture-moderate groups in bladder capacity, maximum bladder pressure, and bladder compliance (Figure 5B–D).

In the cystectomy experiment, for the GSF-mild group, bladder capacity was 786.82 ± 52.71 μL, maximum bladder pressure was 36.55 ± 5.35 mmHg, and bladder compliance was 21.72 ± 1.74 μL/mmHg. In the suture-mild group, bladder capacity was 747.15 ± 29.49 μL, maximum bladder pressure was 37.64 ± 2.88 mmHg, and bladder compliance was 19.96 ± 1.88 μL/mmHg. No statistically significant differences were found between the GSF-mild and suture-mild groups in these parameters. In the GSF-moderate group, bladder capacity was 630.22 ± 27.79 μL, maximum bladder pressure was 35.79 ± 4.34 mmHg, and bladder compliance was 17.77 ± 1.59 μL/mmHg. In the suture-moderate group, bladder capacity was 582.92 ± 45.62 μL, maximum bladder pressure was 37.20 ± 2.87 mmHg, and bladder compliance was 16.16 ± 1.38 μL/mmHg. No statistically significant differences were observed between the GSF-moderate and suture-moderate groups in bladder capacity, maximum bladder pressure, and bladder compliance. In the GSF-severe group, bladder capacity was 491.95 ± 22.31 μL, maximum bladder pressure was 36.50 ± 3.14 mmHg, and bladder compliance was 13.54 ± 0.90 μL/mmHg. In the suture-severe group, bladder capacity was 454.74 ± 25.15 μL, maximum bladder pressure was 36.28 ± 1.91 mmHg, and bladder compliance was 12.10 ± 0.99 μL/mmHg. No statistically significant differences were observed between the GSF-severe and suture-severe groups in terms of bladder capacity, maximum bladder pressure, and bladder compliance (Figure 5E–G).

### 3.6. Histological Evaluation

**Figure 6:**
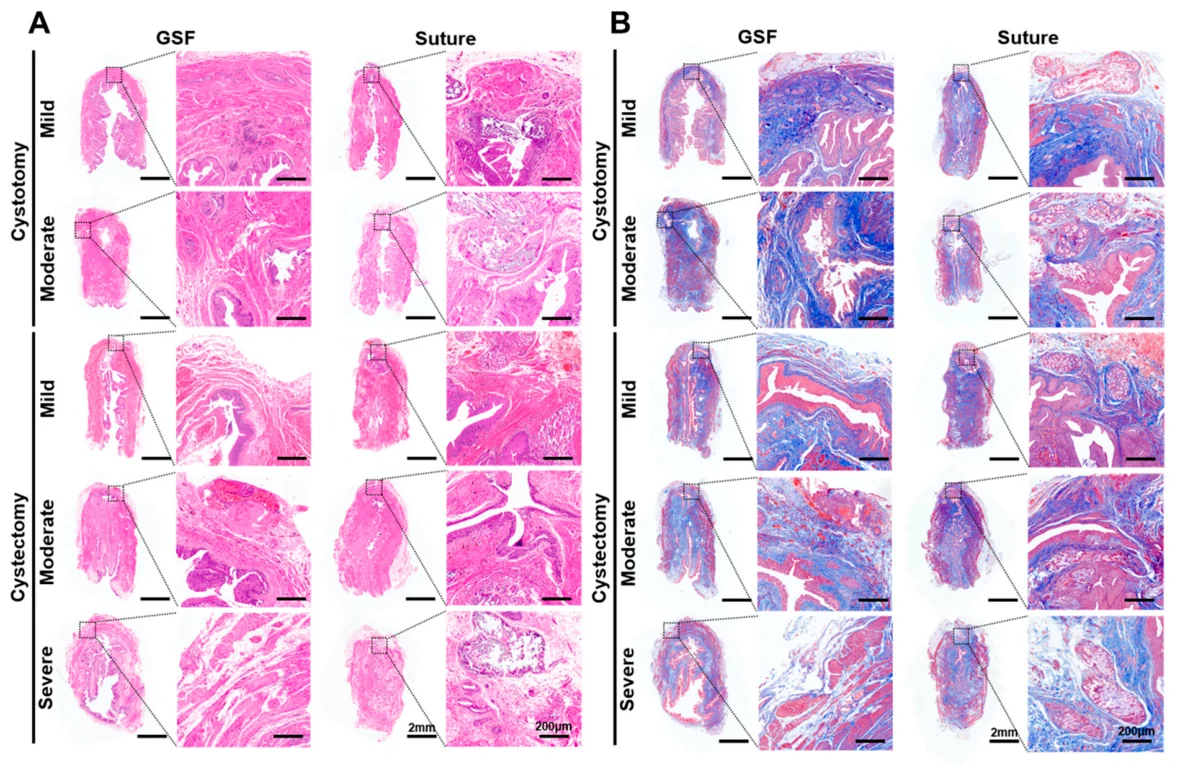
Histological staining of regenerated bladder wounds. (**A**) H&E staining of repaired bladder tissues. (**B**) Masson trichrome staining of repaired bladder tissues.

H&E and Masson staining were performed on tissue sections collected 4 weeks after surgery (Figure 6). In the cystotomy experiment, the bladder wound was clearly visible. In some GSF-treated groups, a small amount of hydrogel adhesive remained in the tissues and on the bladder wall surface, whereas the suture group exhibited incomplete degradation of sutures. Urothelial regeneration was complete across all groups. In the GSF group, a significant amount of loose collagen fibers and orderly proliferated smooth muscle were observed at the repair site. In contrast, the suture group showed denser collagen fibers with disordered smooth muscle regeneration around the remaining sutures. Histological findings in the cystectomy experiment were similar to those observed in the cystotomy experiment.

### 3.7. Immunofluorescent Staining

**Figure 7:**
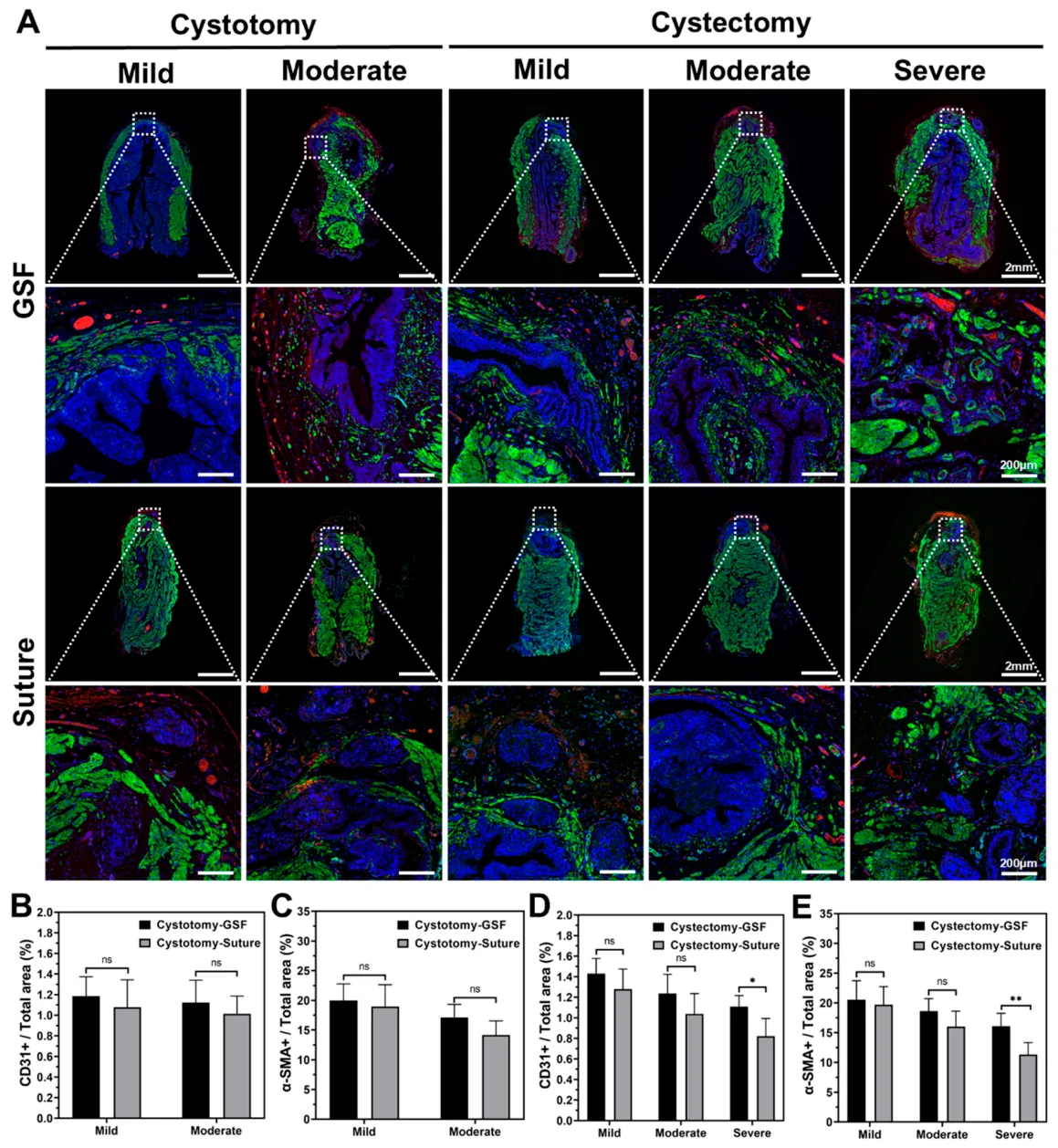
Immunofluorescence assessment of smooth muscle regeneration and angiogenesis in repaired bladder tissues. (**A**) Immunofluorescence staining of α-SMA (smooth muscle, green), CD31 (blood vessels, red), and DAPI (cell nucleus, blue). (**B**,**C**) Quantitative analysis of CD31 and α-SMA positive areas in cystotomy experiments. (**D**,**E**) Quantitative analysis of CD31 and α-SMA positive areas in cystectomy experiments. (*p*-values are calculated using the two-way ANOVA followed by Sidak’s post hoc test, ns, *p* > 0.05, * *p* < 0.05, ** *p* < 0.01).

Immunofluorescent staining for α-SMA and CD31, along with quantitative analysis, was performed 4 weeks post-surgery (Figure 7A). In the cystotomy experiment, the α-SMA positive area ratio in the GSF-mild group was 19.98% ± 2.80%, and the CD31 positive area ratio was 1.19% ± 0.19%. In the suture-mild group, the α-SMA positive area ratio was 18.99% ± 3.64%, and the CD31 positive area ratio was 1.08% ± 0.27%. In the GSF-moderate group, the α-SMA positive area ratio was 17.13% ± 2.20%, and the CD31 positive area ratio was 1.12% ± 0.22%. In the suture-moderate group, the α-SMA positive area ratio was 14.16% ± 2.39%, and the CD31 positive area ratio was 1.01% ± 0.17% (Figure 7B,C).

In the cystectomy experiment, for the GSF-mild group, the α-SMA positive area ratio was 20.56% ± 3.17%, and the CD31 positive area ratio was 1.43% ± 0.15%. In the suture-mild group, the α-SMA positive area ratio was 19.73% ± 3.02%, and the CD31 positive area ratio was 1.28% ± 0.20%. For the GSF-moderate group, the α-SMA positive area ratio was 18.64% ± 2.09%, and the CD31 positive area ratio was 1.24% ± 0.19%. In the suture-moderate group, the α-SMA positive area ratio was 16.04% ± 2.58%, and the CD31 positive area ratio was 1.04% ± 0.20%. In the GSF-severe group, the α-SMA positive area ratio was 16.08% ± 2.18%, and the CD31 positive area ratio was 1.11% ± 0.11%. In the suture-severe group, the α-SMA positive area ratio was 11.31% ± 2.01%, and the CD31 positive area ratio was 0.82% ± 0.17%. Notably, in the severe cystectomy experiment, the α-SMA and CD31 positive area in the GSF-severe group was significantly greater than that in the suture-severe group (*p* < 0.05, Figure 7D,E).

## 4. Discussion

This study expands on our previous findings by further characterizing the properties of the GSF hydrogel and conducting an extensive rat model experiment to assess the efficacy of the GSF hydrogel adhesive. The GSF hydrogel exhibits injectability, mechanical strength, good biocompatibility, and bioadhesive properties. The rat bladder repair experiments indicate that GSF hydrogel adhesive offers an effective repair for mild and moderate bladder ruptures as well as partial bladder defects. However, both suturing and adhesive strategies demonstrate limited therapeutic efficacy against severe bladder rupture injuries. Post-mortem examination revealed urine leakage adjacent to the urethra in the GSF group, which was attributed to severe local tissue damage and insufficient wound apposition in anatomically complex regions. In comparison, no urine leakage was detected in the suture group. Nevertheless, sutures placed in overly close proximity to the bladder trigone caused iatrogenic injury to the ureters and urethra, which probably accounted for the mortality of these rats. Therefore, the GSF hydrogel may not be suitable for the repair of severe bladder trauma, especially for injuries adjacent to the urethra. For injuries adjacent to the bladder trigone, meticulous surgical manipulation is strongly recommended to prevent damage to vital surrounding structures. In clinical practice, tight suturing to ensure the closure of cavity organs can be time-consuming. Although surgical staplers have improved the shortcomings of sutures, complications such as postoperative bleeding, edge ulcers, and anastomotic leakage still persist [12,13]. Compared with suturing, the GSF hydrogel achieves reliable bladder closure while significantly shortening operation time, demonstrating distinct operational advantages for the repair of mild-to-moderate bladder ruptures and partial bladder defects.

Cavitary organs, such as the bladder, function as containers for liquid storage, and leakage can have serious consequences. Therefore, adhesives used in such repairs must possess strong adhesive properties, mechanical strength, sealing ability, and wet adhesion capabilities. These attributes are essential for effective repair [14]. Silk fibroin provides excellent mechanical properties and can withstand significant traction during bladder repair and reconstruction [15]. SilMA is a methacrylate-modified silk fibroin that retains the superior mechanical properties and good biocompatibility [16]. Therefore, it was selected as the primary component of the GSF hydrogel. Numerous studies have shown that GelMA hydrogels can promote cell adhesion and migration, which is facilitated by the hydrophilic network structure, appropriate porosity, and the presence of the arginine-glycyl-aspartic acid peptide, a key cell adhesion site [17,18,19]. However, hydrogels composed of SilMA and GelMA still lack sufficient mechanical tensile strength for bladder adhesion repair. Zhang et al. demonstrated that 5% (*w*/*v*) F127DA enhances the interactions among the hydrogel components and improves the mechanical stability of the hydrogels [20]. The results demonstrate that the incorporation of F127DA remarkably improves the mechanical strength of the GSF hydrogel. F127DA molecules induce intermicellar and adjacent intermicellar shell crosslinking to form a dynamic noncovalent polymer network, which serves as energy-dissipating giant crosslinking junctions and markedly enhances the mechanical stability of hydrogels [21]. A self-designed device was used to verify the reliable sealing performance of the bladder under dynamic wet conditions. After bladder surgery, catheters are routinely indwelling to keep the bladder empty, and perfusion with 240 mL of urine sufficiently proves the good sealing and wet adhesion performance of the GSF hydrogel. However, due to its inherent limitations, this device still cannot fully replicate the peritoneal environment in vivo. Supplementary investigations incorporating more physiologically relevant models will be prioritized in our future work. The diffusion of the GSF hydrogel precursor solution through tissue interstitial spaces, coupled with rapid cross-linking via light irradiation, promotes enhanced wet adhesion and sealing.

The use of biomaterials in bladder injury repair may lead to complications such as stone formation due to the immunogenicity and degradation of these materials [22,23]. Ultrasound examination confirmed that both the GSF and suture groups exhibited well-filled bladders. Histological examination revealed that the bladder tissue in the repair area remained homogeneous, without fibrosis or calcification. This is attributed to the good biocompatibility of the GSF hydrogel and its degradation properties in the urine environment. GelMA and SilMA hydrogels, derived from natural organisms, are known for their excellent biocompatibility. These materials are commonly used in cell culture and have been shown to promote skin lesion healing due to their water retention and sealing properties [24,25]. F127DA, a hydrogel with photo-crosslinking capabilities resulting from F127 modification, also exhibits strong biocompatibility [21,26]. We investigated the degradation curve of the GSF hydrogel in the urine environment for 2 weeks. It was found that the GSF hydrogel degraded stably and slowly within 10 days, followed by an accelerated degradation rate after 10 days. This degradation characteristic enables the hydrogel to exert an adhesion effect in the early stage, and the rapid late-stage degradation is consistent with tissue repair. Histological examination at 4 weeks revealed only a minimal amount of residual hydrogel in the tissue interstices, suggesting that the hydrogel had been basically completely degraded by 4 weeks. Nevertheless, the tissue inflammatory response caused by GSF degradation remains unclear, which is relevant to the biosafety of the material. Further studies will be conducted to explore the underlying inflammatory mechanism of GSF. Additionally, the suture group exhibited significant omental adhesion, while only slight adhesion was noted in the GSF group. Urological surgeries typically rely on surrounding tissues to prevent urine leakage and promote wound healing [27]. However, postoperative adhesions can be influenced by several factors, and severe abdominal adhesions may lead to complications such as intestinal obstruction [28]. The GSF hydrogel, with its swelling capacity and excellent sealing properties, not only absorbs tissue fluid and blood but also isolates the surrounding tissue compartments, thereby reducing postoperative adhesion.

The bladder wall consists of the urothelium, smooth muscle, and serosa layers. The function of the bladder primarily relies on the urothelium and smooth muscle. Histological examinations of the bladder wall microstructure revealed that the GSF group outperformed the suture group in terms of collagen fiber organization, smooth muscle regeneration, and vascular development. The inclusion of GelMA in the GSF hydrogel assists in promoting cell regeneration, migration, and angiogenesis during bladder repair. In the suture group, the incomplete degradation of the surgical sutures resulted in a disordered bladder wall structure, dense collagen fibers, inflammatory cell infiltration, and the formation of tissue scars. The regeneration and reconstruction of blood vessels are critical for tissue repair. An et al. demonstrated that combining GelMA hydrogel, adipose-derived mesenchymal stem cells, and bladder acellular matrix to create a double-layer bladder patch facilitated the regeneration and vascularization of bladder tissue [29]. In bladder reconstruction, the appropriate porosity of the hydrogel is conducive to the reconstruction of blood supply, which is crucial for the regeneration and repair of bladder smooth muscle.

Immunofluorescence staining revealed more pronounced regeneration of bladder smooth muscle and blood vessels. Quantitative analysis of the α-SMA and CD31 fluorescence areas indicated that the α-SMA fluorescence area in the GSF-severe group was significantly larger than in the suture-severe group. This difference was observed only in the cystectomy experiment. Wound healing occurs in three distinct phases—inflammation, proliferation, and tissue remodeling—which are well documented in skin healing. The healing process of bladder wounds mirrors that of skin [30]. Mechanical tension at the wound site promotes scar formation. Under mechanical tension, fibroblasts transition to a fibrotic phenotype via tension-sensing pathways, triggering fibrosis and subsequent scar formation. Various biomaterials with mechanical regulation properties can play a beneficial role in preventing scarring [31,32]. In the case of large bladder resections, wound tension directly impacts the regenerative repair of the bladder wall. GSF hydrogel alleviates this tension while promoting cell adhesion and migration, especially in larger wounds. In mild and moderate bladder injuries, wound tension has a lesser negative impact, thus diminishing the positive effects of GSF hydrogel.

Given its role in storing and expelling urine, the recovery of bladder function is crucial. Urodynamic examination serves as an effective method for evaluating bladder physiological function. In this study, no significant differences were observed between the GSF and suture groups regarding bladder capacity, maximum bladder pressure, or bladder compliance when comparing injuries of the same severity, indicating that both GSF hydrogel and sutures offer comparable repair efficacy. The residual bladder wall may have compensated for partial bladder function. The regeneration of smooth muscle and nerves after bladder injury is critical for restoring bladder function [33,34]. In the cystectomy experiment, bladder compliance in the GSF group tended to be higher than in the suture group at the same injury severity, although the differences were not statistically significant. From a clinical perspective, combined with histological analysis, it can be speculated that GSF hydrogel promotes smooth muscle regeneration and scarless repair, which contributes to improved bladder compliance.

Several intriguing observations emerged during the experiment. For instance, postoperative morphology suggests that the GSF hydrogel may possess anti-adhesive properties, and its excellent sealing ability might help prevent bleeding. Wan et al. developed a multifunctional hydrogel for hemostasis and anti-adhesion based on free radical polymerization and ion coordination, which has shown significant promise in nephron-sparing surgery [35]. The GSF hydrogel may share similar properties, indicating potential for broader applications in preventing adhesions and controlling bleeding. Furthermore, hydrogels are widely recognized as effective drug-delivery platforms [36]. Yang et al. utilized a porcine dermal acellular matrix hydrogel loaded with levofloxacin for intravesical injection, significantly enhancing the treatment effectiveness and targeting in urinary tract infections [37]. Zhao et al. highlighted the beneficial effects of angiogenic factors in bladder injury repair [38]. GSF hydrogel could potentially be loaded with cell growth factors or therapeutic agents to further enhance bladder regeneration and repair.

Despite comparable macro-physiological functional recovery reflected by urodynamic parameters between the GSF hydrogel group and the conventional suture group, GSF hydrogel still possesses distinct advantages from translational perspectives. First, the adhesive can be directly applied and photopolymerized onto the defect in situ, which markedly simplifies operative manipulation and shortens operative duration relative to traditional suture techniques. This technical ease holds great clinical significance for reducing intraoperative trauma, lowering technical barriers for surgeons, and minimizing operation complications [39]. Second, the GSF hydrogel shows clear superiority in reducing postoperative intra-abdominal adhesions. Severe adhesions can cause abdominal pain and intestinal obstruction and increase the technical complexity of potential re-operations, posing a major unmet clinical challenge in urological reconstructive procedures [40]. Beyond macroscopic observations, superior microscopic repair outcomes are seen in GSF-treated defects, characterized by enhanced smooth-muscle regeneration and neovascularization, as supported by elevated α-SMA and CD31 expression. Collectively, these advantages endow GSF hydrogel with unique translational value for bladder injury repair.

This study has certain limitations. Specifically, the effects of GSF hydrogel adhesive have only been demonstrated in a small sample of rats, with no application in larger animals. Biological variability cannot be eliminated. Nevertheless, bladders with varying degrees of injury exhibited high consistency in the overall Efficacy, demonstrating the reliability of the results. Experimental rigor was further ensured by standardizing the animal model and surgical operator. Despite this, the evidence provided strongly supports the potential clinical application of GSF hydrogel adhesives. Future research will focus on large-sample and large-animal experiments to verify the effectiveness and safety of the GSF hydrogel in repairing bladder injuries.

## 5. Conclusions

The GSF hydrogel, composed of GelMA, SilMA, and F127DA, offers fast photo-crosslinking, robust bonding properties, mechanical strength, and an effective sealing capacity. The rat bladder repair experiment demonstrated that GSF hydrogel adhesive provides rapid gelation and excellent wet adhesion. Compared to suturing, GSF hydrogel significantly enhances operational efficiency. It effectively repairs mild to moderate bladder ruptures and partial bladder defects, facilitating structural reconstruction and promoting functional recovery of the bladder.

## Data Availability

The datasets analyzed during the current study are available from the corresponding authors upon reasonable request.

## Supplementary Materials

The following supporting information can be downloaded at: https://www.mdpi.com/article/10.3390/bioengineering13091047/s1, Video S1: Repair of bladder injury by GSF hydrogel adhesives; Video S2: Repair of bladder injury by sutures.

## Abbreviations

The following abbreviations are used in this manuscript:

GelMA	gelatin methacryloyl
SilMA	silk fibroin methacryloyl
F127DA	polyether F127 diacrylate
GS	GelMA-SilMA
GSF	GelMA-SilMA-F127DA
LAP	lithium phenyl (2,4,6-trimethylbenzoyl) phosphinate
SEM	Scanning electron microscope
FTIR	fourier transform infrared spectroscopy
H&E	hematoxylin and eosin
α-SMA	α-smooth muscle actin
